# Supplementary material for: A population study of Toxoplasma gondii in the Amazon region expands current knowledge of the genetic diversity in South America
Source: PLoS Negl Trop Dis. 2024 Dec 23;18(12):e0012737. doi: 10.1371/journal.pntd.0012737 (PMC11698570; doi:10.1371/journal.pntd.0012737)
Supplement: Attachment — Submitted filename: response letter_2_plos.docx [file pntd.0012737.s004.docx]

November 7th, 2024

RESPONSE LETTER

Reviewer #1:

Thank you for your comments and suggestions.

- **Were possible cross reactions taken into account when performing serological assays? Please expand on this.**

Free-range chickens are exposed to many different microorganisms in their environment, with some of them closely related to *T. gondii* (particularly *Sarcocystis* and *Eimeria*). In Brazil, *Eimeria* and *T. gondii* are, in general, highly prevalent in free-range chickens, so co-infections should be frequent. However, even with closely related antigens, it seems specific cross-immunity does not occur, and even between closely related *Eimeria* species cross-immunity is not observed (Rose, 1987; Dubey et al., 1993; Hiob et al., 2017). About *Sarcocystis*, as far as we know, there is no report in chickens from Brazil.

Two comprehensive studies have validated the MAT for serologic diagnosis of *T. gondii* in chickens (Dubey et al., 2016; Schares et al., 2018). Dubey et al. (2016) observed a high negative predictive value of the MAT and Schares et al. (2018) concluded that MAT is suitable to identify seropositive *T. gondii* chickens under field conditions relatively to direct positive detection by mouse bioassay, Magnetic-Capture real-time PCR or real-time PCR on acidic pepsin digested chicken tissues (diagnostic sensitivity was 65.2% and diagnostic specificity was 100%). So, even if some kind of cross-reaction, not yet reported, occurs, it seems it does not interfere with epidemiological results. MAT was the serological test chosen because it is simple, does not require special equipment or conjugates, the antigen is very stable, and many samples can be run at the same time into a plate, which was important during the investigation.

We have included the following sentence in the manuscript about the validation of the serological test used:

Lines 145-146:

“The MAT has been previously validated as a serological test for *T. gondii* diagnostic in chickens [24,25].”

References cited above:

Rose, ME (1987). Immunity to *Eimeria* infections. Veterinary Immunology and Immunopathology 17, 333-343

Dubey et al. (1993). Serologic and parasitologic responses of domestic chickens after oral inoculation with *Toxoplasma gondii* oocysts. American Journal of Veterinary
Research 54, 1668-1672.

Hiob et al. (2017). Experimental *Toxoplasma gondii* and *Eimeria tenella* co-infection in chickens. Parasitology Research 116, 3189-3203.

Schares et al. (2018). *Toxoplasma gondii* infections in chickens – performance of various antibody detection techniques in serum and meat juice relative to bioassay and DNA detection methods. International Journal for Parasitology 48, 751-762.

- **Can authors account for the differences between the number of seropositive chickens and the final number of animals bioassayed in mice? As per the methodology description, tissues from all positive chicken were bioassayed in mice, however the number of bioassays is lower than the seropositive animals. This should be explained in the methodology or results section.**

We agree this point is not clear in Material and methods section. Mostly, we performed bioassays with chickens with MAT titers ≥ 10, with some exceptions, depending on mouse availability for inoculation (in DISCUSSION section, we commented on this at lines 470-473). However, when there were mice remaining in the mouse batch purchased, we also performed bioassays with chickens with titer 5. We have reformulated the sentence in the manuscript:

Lines 152-156:

“The brain and heart homogenates of seropositive chickens with MAT titers ≥ 10 (see Table 2 for exceptions) were individually bioassayed in Swiss Webster mice for *T. gondii* isolation using 3 to 5 mice per chicken. Bioassays from chickens with serologic titer 5 were performed only partially, if there were mice available for inoculation.”

- **L505: Recent studies have shown that the CS3 marker is actually not a good predictor of Toxoplasma virulence in mice (e.g. doi: 10.1186/s13567-021-00953-7). This should be acknowledged and addressed in the discussion.**

We agree our discussion was very incomplete about the role of CS3 as a predictive marker of *T. gondii* virulence in mice, so we have changed it, with a review and more precise discussion on this subject. We think there is a great misunderstanding on this subject since the studies by Pena et al. (2008), which found, statistically, a strong association, but not an exclusive association, if you analyze each isolate individually:

Lines 565-597:

“Molecular markers could be an alternative to predict *T. gondii* virulence in mice, minimizing the use of this model. In this sense, the CS3 marker was used in the present study to assess virulence in a mouse model, as it was previously reported as strongly linked to acute virulence in this model [86]. It was observed that CS3 allelles I and II had a strong association with virulent and intermediate virulent isolates and CS3 allele III with avirulent isolates. It is necessary to be cautious when comparing these results with others because, as exposed above, there is a lack of standardization in the isolation methods related to the route of inoculation (subcutaneous or intraperitoneal), mouse lineage (Balb-c or Swiss), dose (protocols with known doses and unknow doses), stage of the parasite inoculated (bradyzoites or tachyzoites) and criteria for virulence, all of which are factors that could interfere with the results.

Results of *T. gondii* mouse virulence based in the CS3 I, II and III alleles in the present study are comparable to those from Brazilian isolates (a total of 194 isolates from diverse hosts analyzed) reported in other studies [9,38,50,55,61,62,77,87–90]. For other studies from Brazil (a total of 179 isolates from diverse hosts analyzed), there is a major concordance for CS3 alleles I and II as virulent or intermediate but CS3 allele III showed avirulent, virulent or intermediate virulences [40,55,83,84,91,92]. Also, two studies from Spain showed that three clonal Type III isolates (CS3=III) were mouse virulent [93,94]. It has been suggested that factors such as different proteins (e.g. pseudokinases, kinases, polymorphic dense granule protein) secreted into the host cell during invasion would have a role in the virulence of isolates with CS3 allele III [40]. It is noteworthy that nine clonal Type II (CS3=II) sheep isolates from Spain were reported as avirulent for mice [94], corroborating previous studies in Brazil [40,60,78,87], where this archetypal genotype has a very low circulation, which suggests a different modulation of virulence for this genotype. So, overall, the present study, and most of the previous studies in Brazil suggests that, when examining a set of samples, isolates with CS3 alleles I and II have great probability to be virulent or intermediately virulent in mice, but in Europe, where archetypal type II predominates, it seems this conclusion does not apply. It is important to point out that no association of CS3 allele type with clinical manifestations related to human congenital toxoplasmosis was previously reported [40].”

Lines 602-604:

“The definitive role of molecular markers in determining the exact virulence in mice or in clinical human manifestations of toxoplasmosis has yet to be determined.”

Reviewer #2:

Thank you for your comments and suggestions.

**There a few points relating to the murine infections that were not clear:**

**1) Since the starting dose of T. gondii is unknown is it possible that the difference in virulence is not due to the isolate but due to initial dose? If this is the case could the authors comment on this possibility in the discussion.**

There are many factors that affect the virulence in mice including the stage of the parasite inoculated, route, types of mice used, the strain of the parasite and dose. But these differences have not been comparatively assessed yet. In our study, we classified the isolates based on certain criteria, considering we did not wait for the mice to die, because we believe it is not ethically acceptable anymore, especially considering a large number of isolates, as in this case. To make it clearer, we have made some changes in the manuscript:

In RESULTS section:

Lines 287-289:

“…according to these parameters, these isolates were biologically estimated to be virulent, non-virulent, and intermediately virulent, respectively (Table 3)”.

In Table 3:

Lines 298-299:

^f^VIR = virulent: acute sickness observed in more than 50% of the infected mice within 30 days p.i.; **NVIR** = non-virulent: no clinical signs observed in the infected mice; INT = intermediate virulence: intermediate behavior.

Also, in DISCUSSION section we have written this paragraph below to comment on this:

Lines 541-564:

“Phenotypically, *T. gondii* isolates in mice are usually classified as virulent, non-virulent, or of intermediate virulence. This classification can be defined in a second round of mouse (normally Swiss or Balb-c lineages) inoculations (normally intraperitoneal), after isolation, based on the percentage of mortality of mice infected with different parasite (normally tachyzoites) doses (usually 10^0^, 10^1^, 10^2^, 10^3^) [82].This system can be very expensive, time and space consuming when applied to a high number of samples as in the present study. Alternatively, although less reliable, virulence could be defined during the isolation process, also based on mouse mortality, when unknow doses of parasites (normally bradyzoites from digested tissues from chronic host infections) are inoculated (generally subcutaneously) in mice, as in most of the isolation studies in Brazil. The cumulative mortality parameter to define virulence in mice can be considered ethically questionable nowadays. In the present report, virulence definition was based on other criteria, using an endpoint for euthanasia [27]; other studies in Brazil also adopted endpoints to mouse euthanasia [83,84]. Most isolates exhibited a virulent phenotype in mice, as expected for isolates from South America [85]. It is important to remark that many factors can interfere with mouse virulence including the stage of the parasite inoculated, route, dose, lineages of mice used in the bioassays and the strain of the parasite [1]. The number of mice inoculated in each bioassay group could also interfere with virulence interpretation, particularly if only one mouse gets infected as occurred in some groups in the present study. In summary, there is no standardization on mice virulence assays in the current literature, making result comparisons difficult.”

2**) In the Materials and Methods it states:
The inoculated mice were considered “infected” with the parasite when tachyzoites or tissue cysts were present in the lungs and brain, respectively. (Lines 159-161). I just wanted to confirm that this is what “infected” means in Table 3 e.g. for TgCkBrAC3 where it says Sick/Infected 0/3 all three mice had tachyzoites/tissue cysts but did not display sickness behaviour? If this isn’t what “infected” means here could the authors please clarify.**

Exactly, e.g. “sick/infected 0/3” means three mice had stages of the parasite in their tissues, but none of them had clinical signs related to the disease. We have included a legend in the Table 3 to make it clearer when analyzing solely the table:

Line 294:

“^c^Mice were considered infected with the parasite when tachyzoites or tissue cysts were present in their tissues using direct microscopic examination.”

**3) Potentially this is me not understanding the table correctly but for some strains there is only 1 mouse sick out of 1 mouse infected, whereas others have 3. If only 1 mouse is infected is it really possible to identify intermediate virulence? Again, I think it could be commented upon in the discussion that this is based on very small numbers and unequal group sizes so predictions of virulence should be made with caution**.

To choose primary isolation of the parasite to define virulence is not so reliable because the number of parasites is not known, but to perform classic virulence studies, posteriorly inoculating other mice with a series of known doses of tachyzoites is not feasible when working with a large set of samples as in the case of this study, so, here, virulence was a primary estimative based on criteria adopted for this study. We have added a paragraph discussing on this matter (please, see the answer to question 1).

Reviewer #3:

Thank you for your comments and suggestions.

**1.1-Line 348 “:.... genotypes in Brazil were identified for the first time in the Brazilian Amazonr”.  Review Amazon r?**

We have revised this typing mistake:

Lines 364-365:

“… in the Brazilian Amazon region.”

**Lines 352-355: “These genotypes included 81 genotypes (from 218 isolates) previously reported in the Southeastern region of Brazil, 52 genotypes (116 isolates from this study and 19 from previous studies) in the Northern region of Brazil”.  Which previous studies are you referring? Please cite the reference(s).**

We have added this information in the manuscript:

Lines 368-371:

“These genotypes included 81 genotypes (from 218 isolates) previously reported in the Southeastern region of Brazil [6,9,27,36–68], 52 genotypes (from 116 isolates from this study and 19 previously reported [6,21,44,61,69,70]) in the Northern region of Brazil…”

**1.2-Lines 361- 365: “Most of the genotypes observed in the Amazon region in this study and in other studies in the Northern region of Brazil were clustered in Group 1 (29 genotypes, 44 isolates), and most of the genotypes described in the Southeastern region of Brazil were clustered in Group 5 (51 genotypes, 230 isolates)”. Also, include the reference (s) related to “from other studies”.**

The references from other studies have been included:

Lines 378-382:

“Most of the genotypes observed in the Amazon region in this study and from other studies in the Northern region of Brazil [6,21,44,61,69,70] were clustered in Group 1 (29 genotypes, 44 isolates), and most of the genotypes described in the Southeastern region of Brazil were clustered in Group 5 (51 genotypes, 230 isolates).”

**1.3-Lines 418-419:“.... (103 isolates from 419 the present study and 47 isolates from previous studies.....” Change to ....103 isolates from 419 from the present study and 47 isolates from previous studies...”.**

We have observed that this paragraph was already correct in the manuscript, so nothing has been changed (this “419” is not in the manuscript).

Lines 436-440:

“The value of *D* for the entire *T. gondii* population used in the present study (116 isolates) was 0.872, that for the Northern region of Brazil (103 isolates from the present study and 47 isolates from previous studies in Pará, Rondônia, and Amazonas states was 0.908, and that for the Southeastern region of Brazil (90 genotypes, 341 isolates) was 0.961.”

**2.1-Lines 438-446    Brazil, Colombia, and Argentina are hotspots for T. gondii genetic diversity 437 in South America. In Brazil, this knowledge comes particularly from isolates obtained from the Southeastern region; however, when describing the circulation of T. gondii genotypes in the country, it is important to note that Brazil is the fifth   largest country in the world and the largest country in South America and Latin  America occupying almost 50% of South American territory; São Paulo city, in  the Southeastern region is separated from Manaus city, in the Northern region, by almost 4,000 km. Additionally, the human population is not evenly distributed across the country, with high concentrations in the Southeast and along the coast. Therefore, most of the information regarding T. gondii diversity in Brazil has been focused on the most populated and urbanized areas”.
Discussion suggested: From the perspective of One Health,  what would be the impact of these findings for the environment and the population living in the studied areas?**

We have added this paragraph to discuss the importance of our findings to the environment and the population living in the studied areas:

Lines 670-680:

“From the perspective of One Health, the importance of these findings for the population living in the region has been observed in studies with human populations in urban and rural areas [21,99] and in some studies also describing outbreaks of clinical toxoplasmosis [100,101]. Also in the Amazon region, in French Guiana and Suriname, description of very severe clinical toxoplasmosis, even in immunocompetent patients, were described, and called "Amazonian Toxoplasmosis" [12,17]. However, there are still few studies when observing the vast area that makes up the Amazon territory and the great diversity of cultures and wildlife that live in this region, which makes it difficult to implement control measures, whether animal or environmental, but point to the need to implement strategic measures with the human populations.”

**2.2-Lines 479-484 –“Archetypal clonal Types I (#10), II (#1 and #3), and III (#2) were not observed. These classical types exhibit low frequencies in the Brazilian territory, with a small number of Type I and Type II strains circulating in the Southern region  of Brazil [42–45], Type II variant (#3) particularly on Fernando de Noronha Island  [3,46], and some Type III strains were isolated in all regions of Brazil except for the North [3,29]”.
Discussion: How do the authors explain the differences in prevalence and distribution of these strains found in the North versus Southern region of Brazil?**

We have added the following paragraph in the manuscript to discuss on this subject:

Lines 516-528:

“Studies on global diversity of *T. gondii* has showed that the world trade, human migration and slave trade through maritime route had a remarkable role in the spread of the archetypal genotypes from Europe to other continents during colonial time through transportation of invasive mice, rats and cats [4,71,80]. So, in Brazil and other countries of South America this event has probably occurred between 16th and 19th centuries with the territory colonization by Portugal and Spain, respectively. However, in an environment already populated with hundreds of strains, there was no space for expansion, probably explaining why archetypal genotypes are so limited in those territories. In Brazil, the colonization started in the coast, particularly in the south and southeast, so this would have preserved the northern region from initial contact with these new strains because of the geographical distance together with undeveloped means of terrestrial transportation.”

**2.3- Lines 489 – 498: “Samples from Guyana, Venezuela, Bolivia, and Peru were collected from municipalities bordering Brazil; however, the eight genotypes reported here (including four new genotypes) were different from those from the five Brazilian  states searched (Fig 3). Three of them (#12, #95, and #123) have never been  reported in Brazil. Genotype #12 has been previously reported as the most  prevalent genotype in Guyana [3,37], and it was also observed in the present study, likely indicating its high circulation in this country. Although few isolates were analyzed in the present study, the results contribute to our knowledge of the distribution and diversity of T. gondii genotypes in other South American countries”.
Discussion: What are the possible explanation(s) for the results described above such as the differences between the foreign municipalities bordering Brazil and the North of Brazil?**

To discuss on these differences, we have added the following paragraph:

Lines 535-537:

“These differences may just reflect both the high diversity in the vast biome studied, where borders are merely administrative separation, and the limited number of samples collected.”

**2.4- A discussion of the strengths and limitations of the study needs to be included.**

We agree it was necessary to include a discussion on the strengths and limitations of our study. The following paragraph on this matter has been included:

Lines 467-481:

“Therefore, most of the information regarding *T. gondii* diversity in Brazil has been focused on the most populated and urbanized areas. To fill this gap, the largest collection of *T. gondii* isolates (116 isolates from chickens) was obtained from the Amazon region that is the most important area of the remaining forest in South America. Additionally, this is the largest collection of new *T. gondii* PCR-RFLP genotypes recently described in a single study (20 new genotypes), thus contributing to the knowledge on the genetic diversity of *T. gondii* worldwide. On the other hand, in the present study, the wild animal population could not be assessed because of the limitations of resources, legal permissions, logistic in extremely remote areas, and, particularly, safety, so it is almost certain that the present study could not still picture the complete diversity of alleles circulating in the Amazon region, because it is known that intermediate and definitive host diversity in an environment can contribute significantly to the diversification of the parasite through the establishing of a wild cycle [4], as demonstrated in French Guiana [8].

To the Editor:

About the additional instructors on the manuscript:

**Figure Files:**

Figures were uploaded to the PACE on 09/18/2024 (11:44-11:46am). No image problems were found. Pace adjustments were made in Fig 3 (the new version was inspected, and image clarity and content were not affected).

**Data Requirements:**

All data used to draw the conclusions outlined in the manuscript are included within the body of the manuscript and in the three files uploaded as supporting information.

**Reproducibility**

The laboratory protocols used in the research are standard in the literature and are described in the body of the manuscript and in the references cited in Materials and Methods.
